# Evaluation of an Extract Derived from the Seaweed *Ascophyllum nodosum* to Reduce the Negative Effects of Heat Stress on Broiler Growth and Stress Parameters

**DOI:** 10.3390/ani13020259

**Published:** 2023-01-12

**Authors:** Gregory S. Archer

**Affiliations:** Department of Poultry Science, Texas A&M University, College Station, TX 77843, USA; garcher@tamu.edu; Tel.: +1-979-845-4319

**Keywords:** broiler, stress, growth, seaweed

## Abstract

**Simple Summary:**

Reducing the negative effects of heat stress on broiler growth and welfare is a goal of the poultry industry. The use of feed additives to obtain this goal has garnered increased attention in recent years. Previous research has illustrated that the inclusion of an extract derived from the seaweed *Ascophyllum nodosum* can lower corticosterone and body temperature in several species. However, it is not known whether this extract would be able to alleviate the negative impacts of heat stress on broiler chickens.

**Abstract:**

Heat stress is one on the main welfare issues that broiler chickens face and it can lead not only to decreased welfare but production as well. The seaweed *Ascophyllum nodosum* has demonstrated the ability in several species to decrease body temperature and affect immune function. To determine whether adding an extract of this seaweed into the diet of broiler chickens would decrease the negative effects of prolong heat stress on broiler growth, a study was conducted. Broilers were fed a control diet with the seaweed extract added at a rate of 0.5 kg/metric ton of feed throughout a 42-day growout or just a control diet. Half of each feed treatment was exposed to two weeks of heat stress (35 °C for 16 h/day) starting at d28 and continued until the end of the trial. Therefore, there were four treatments: a control non-stressed (CNS), control heat stressed (CHS), seaweed-supplemented non-stressed (SWNS), and seaweed-supplemented heat stressed (SWHS). To determine stress susceptibility, the following measures were collected: bilateral asymmetry (ASYM, *n* = 60), heterophil to lymphocyte ratios (HL, *n* = 24), plasma heat shock protein 70 (HSP70, *n* = 24) and plasma corticosterone concentrations (CORT, *n* = 24). Feed conversion, uniformity and weight gain were also determined. The CHS birds had higher (*p* < 0.05) CORT, ASYM, HSP70 and HL than the CNS, SWNS and SWHS birds. The CNS and SWNS birds did not differ (*p* > 0.05) in body weight at d42 but they were both heavier (*p* < 0.05) than in both the heat-stressed treatments. Furthermore, the CHS weighed less (*p* < 0.05) that the SWHS birds. The non-heat-stressed treatments did not differ (*p* > 0.05) from each other in FCR, however the two heat-stressed treatments did differ (*p* < 0.05) from each other in FCR, with the SWHS birds having better FCR than the CHS birds. Heat stress affected bird uniformity with non-heat-stressed treatments having more (*p* < 0.05) uniformity of body weights within a pen than the heat stress treatments. These results demonstrate that adding this seaweed extract to the feed of poultry can reduce their stress during a prolonged heat stress event, though it had no effect on growth or feed conversion. This feed additive could be used to improve the welfare of poultry during heat stress events.

## 1. Introduction

*Ascophyllum nodosum* is a species of brown seaweed, which is found growing in the littoral zone of the North Atlantic Ocean, and it contains various bioactive polysaccharides such as laminarin, fucose containing polysaccharide, and alginates that are beneficial to health and growth [1]. *Ascophyllum nodosum* is the most widely researched seaweed species used for agricultural purposes [2]. Many of those studies have investigated utilizing it or an extract of it in the feed of agricultural animals. The majority of the previous research has focused on mammals, and specifically ruminants and swine. Sharp [3] stated that *Ascophyllum nodosum* can be used at rates up to 5% in diets of poultry, sheep, cattle, pigs, and horses, as it contains trace elements and vitamins important for growth. Bonos et al. [4] concluded that *Ascophyllum nodosum* could be a potential ingredient in poultry feed, as it had no adverse effects on performance, fatty acid profile or lipid oxidation in their investigations.

The positive effects of feeding *Ascophyllum nodosum* to agricultural animals include lowered *Escherichia coli* in the gut of steers and lambs [5]. An extract of *Ascophyllum nodosum* at 0.5 kg/metric ton of feed demonstrated a beneficial effect in reducing the cecal bacterial load in chicks colonized with *Campylobacter jejuni* [6]. Turner et al. [7], has observed increase weight gain in pigs in response to inclusion of *Ascophyllum nodosum* at 20 kg/metic ton of feed. In a study involving broiler chickens [8], *Ascophyllum nodosum* enhanced growth when fed at 30 kg/metric ton of feed. Other species of seaweed have also been observed to increase feed intake, increase carcass weight, and decrease digestive tract fill in livestock [9].

*Ascophyllum nodosum* has been thought to play a role in the bioavailability of trace minerals, vitamins, and/or antioxidants, and alteration of digestibility [10]. Furthermore, *Ascophyllum nodosum* is composed of 10% fucoidan, which is a biologically active compound [11]. Fucoidan has demonstrated the ability to boost cellular immunity and gut microbiota [12], while also decreasing the classic complement system [13], a component of humoral immunity. In addition, *Ascophyllum nodosum* has anti-viral, amplified phagocytic activity and anti-bacterial activity [14,15,16].

Cole [17] demonstrated that *Ascophyllum nodosum* at 20 kg/metric ton of feed alleviated the magnitude of transport stress by minimizing the disruption of electrolyte balance. An extract of the seaweed *Ascophyllum nodosum* has been observed to lower the core body temperature of cattle in hot weather and also stimulate a greater core body temperature in cold weather [2,14,18,19]. Archer et al. [20,21] also observed decreased body temperature during transport or forced walking stress in sheep fed an extract of *Ascophyllum nodosum.* Archer et al. [20,21] also observed decreased basal cortisol and in response to transport stress. Furthermore, *Ascophyllum nodosum* has been demonstrated to decrease the effects of heat stress in cattle [18], sheep [22] and laying hens [23].

The ability of *Ascophyllum nodosum* to improve immunity, reduce stress susceptibility, lower body temperature, and improve growth make it a possible useful tool to mitigate the negative effects of heat stress on broilers. Therefore, the objective of this study was to evaluate supplementing broiler feed with an extract derived from *Ascophyllum nodosum*. It is hypothesized that the supplementation will reduce stress in improve mitigate losses to weight gain and feed conversion in broilers subjected to prolonged heat stress.

## 2. Materials and Methods

### 2.1. Animals and Husbandry

A total of 480 male Cobb 500 broilers were used in this experiment. Birds were equally housed at 20 birds per pen across a total of 24 pens (0.91 m × 2.74 m) over two separately environmentally controlled rooms (12 pens per room). Birds were randomly assigned to each pen; however, initial pen weights were equalized (0.79 kg). Each pen was lined with used pine shavings equipped with one bell feeder and nipple drinking system. Pens were blocked within and the treatments were assigned at random to one of two dietary treatments: control diet (C) (Table 1. Based on Cobb recommendations) or control diet with a seaweed extract (Tasco, Acandian Seaplants Limited, Dartmouth, NS, Canada) added at a rate of 0.5 kg/metric ton of feed. The seaweed extract was added on top of fished diets prior to pelleting and the control diets had equal amounts of sand added prior to pelleting. Birds were fed a three-phase diet consisting of a starter (Days 0–14, crumble), grower (Days 15–28, pellet), and finisher (Days 29–42, pellet). Birds were raised to 42 days of age. Room temperature was maintained at 35 °C for 7 days prior to the start of the experiment, then at 31 °C for the following 8 days. Week 2 temperature was maintained at 29 °C and then allowed to decrease 2.8 °C each week until the ambient temperature was reached. One room of pens consisting of half of each feed treatment was exposed to two weeks of heat stress (35 °C for 16 h/day) starting at d28 and continuing until the end of the trial. The birds were managed according to the guidelines outlined and approved by the Texas A & M University animal care and use committee (IACUC 2016-0358).

### 2.2. Stress Measures

On d42, blood samples were collected from 4 birds per pen. The area around the wing vein was sanitized with 70% alcohol, and then 2 mL of blood was collected from each bird, and a drop was used to prepare a blood smear slide. The remaining blood was injected into a vacutainer (BD 368,056, BD, Franklin Lakes, NJ, USA). Vacutainers were temporarily stored in an ice bath prior to transport to the lab for further processing. Once all samples were collected, the vacutainers were spun down in a Beckman GS-6R centrifuge (Centrifuge 5804, Eppendorf, Hamburg, Germany) for 15 min at 4000 rpm to separate the cells from the plasma. The plasma was drawn into 2 mL micro-centrifuge tubes and stored at −19 °C until further analysis. The blood-smear slides were stained using a hematology staining kit (Cat# 25,034, Polysciences Inc., Warrington, PA, USA), then air-dried.

Plasma corticosterone and heat shock protein 70 concentrations were measured using a commercially available ELISA kits (Corticosterone: Enzo Life Sciences, ADI-901–097, Farmingdale, NY, USA; HSP70: Enzo Life Sciences, EKS-715, Farmingdale, NY, USA). Absorbance was measured using a microplate absorbance reader (Tecan Sunrise, Tecan Trading AG, Männedorf, Switzerland) and analyzed using the Magellan Tracker software program (Magellan Standard, Tecan, Männedorf, Switzerland). Inter and Intra assay %CV were less than 5%.

Heterophil to Lymphocyte ratios (HL) were determined by examining the stained blood smear slides using microscopy (89404-886, VWR International, Radnor, PA, USA) to count the numbers of both heterophils and lymphocytes until the total observed number reaches 100 [24].

Physical asymmetry was measured on 20 birds per pen, following the protocol outlined in Archer et al. [25]. Using a calibrated Craftsman IP54 Digital Caliper (Sears Holdings, Hoffman Estates, IL, USA), the middle toe length, metatarsal length, and metatarsal width were measured for both the right and left legs. The composite asymmetry score was calculated by taking the sum of the absolute value of left minus right of each trait, then dividing by the total number of traits. Thus, the formula for this trial would be (|L-R|MTL + |L-R|ML + |L-R|MW)/3 = composite asymmetry score.

### 2.3. Growth, Feed Conversion and Uniformity

The birds in each pen were weighed individually at d 0 and d 42. The feed was weighed before it was added to the feeder in each pen and the residual feed was weighed back on feed changes so that feed intake could be calculated. Feed conversion ratio (FCR) was calculated by dividing the total feed intake per pen by the total body weight gain per pen and was corrected for mortality (mortality was weighed and added to final pen weights so that all growth was captured in the FCR calculation). Uniformity of pen weights was determined by two methods. The first calculated the percent coefficient of variance (%CV) of each pen and secondly it calculated the percent of birds within a pen that were within ±10% of the pen’s average weight.

### 2.4. Statistical Analysis

All data were analyzed using the GLM model (Minitab 21, Minitab, LLC, State College, PA, USA) with main (diet and heat stress) effects and interactions deemed significantly different at *p* ≤ 0.05. Means that were determined to be significant were further separated using the LSD Test. All of the assumptions of GLM were tested (Shapiro-Wilk test for normality and Levene’s test for homogeneity of variance). No transformations were needed to meet the assumptions.

## 3. Results

The data are presented in Table 2 and Table 3. The heat stress resulted in higher CORT, ASYM, HSP70, and HL and reduced BW and uniformity while increasing FCR (*p* < 0.05). The CHS birds had higher (*p* < 0.05) CORT, ASYM, HSP70 and HL than the CNS, SWNS and SWHS birds. Birds, which were fed the seaweed extract, had lower *p* < 0.05) CORT, ASYM, HSP70 and HL compared to the control birds. Average bird weight did not differ (*p* > 0.05) at d28 prior to heat stress (1.41 ± 0.03 kg) and all birds had consumed the same (*p* > 0.05) average amount of feed per day (158 ± 3 g/day). The CNS and SWNS birds did not differ (*p* > 0.05) in body weight at d42 but they were both heavier (*p* < 0.05) than both the heat-stressed treatments. Furthermore, the CHS weighed less (*p* < 0.05) than the SWHS birds. The non-heat stressed treatments did not differ (*p* > 0.05) from each other in FCR; however, the two heat-stressed treatments did differ (*p* < 0.05) from each other in FCR with the SWHS birds having better FCR that the CHS birds. The heat stress affected bird uniformity, with non-heat stressed treatments having more (*p* < 0.05) uniformity of body weights within a pen than the heat stress treatments. Mortality (5.1 ± 0.6%) did not differ between treatments (*p* > 0.05).

## 4. Discussion

It has been demonstrated previously that supplementing animal feed with *Ascophyllum nodosum* could reduce the effects of stressors [2,14,17,20,21]. Extreme changes in environmental conditions or even those above the thermoneutral zone of poultry result in distress. When an animal experiences distress, it results in the activation of the hypothalamic–pituitary–adrenal axis (HPA) and increased plasma corticosterone levels [26,27,28,29]. Furthermore, when a broiler is exposed to stressful conditions, it can lead to decreased feed intake, lower body weight, and decreased feed conversion [30,31]. This makes environmental stressors such as heat stress a major contributing factor to economic loss in the poultry industry [32]. Heat stress can be acute or, such as in this current study, last for an extended period.

Previously, it has been observed [20,21] that *Ascophyllum nodosum* could reduce stress hormones (cortisol) during stressful situations. This current study observed that corticosterone was reduced in broilers fed an *Ascophyllum nodosum* extract compared to those fed a control diet after a two-week cyclical heat stress. Furthermore, heterophil to lymphocyte ratio and asymmetry score were also lower in supplemented birds in this current study. These two measures have been previously associated with reduced stress susceptibility [33,34]. In another study [35], the reduction in corticosterone during heat stress was not observed in broilers supplemented with an *Ascophyllum nodosum* extract. However, in that study, the broilers were only heat stressed for 8 h a day and only for one week. The current study exposed the broilers to 16 h of heat stress a day for two weeks when the birds were much heavier. It has been demonstrated that the effect of heat stress on corticosterone is related to the length of the exposure [36]; therefore, it is likely Akinyemi and Adewole [35] did not heat stress the broilers to the level that this current study did. To support this conclusion, during the current study plasma, the HSP70 was also increased in the control broilers in response to the heat stress, while in the supplemented broilers, it did not. HSP70 is released in response to heat stress to protect cells [37]. Furthermore, the heat stress in this study clearly reduced body weight, increased FCR, and decreased uniformity demonstrating its effectiveness.

All the birds in this current study weighed the same prior to heat stress and had consumed similar amounts of feed to that point. Meanwhile, others [35,38,39,40] have observed the improved weight gain and FCR in poultry in response to heat stress when diets were supplemented with *Ascophyllum nodosum*, which was not observed in this current study. It is possible that is due to the intensity and duration of the heat stress in this current study. It is possible that if the heat stress was shorter and not as intense the *Ascophyllum nodosum,* the supplementation might have been able to mitigate the effects similar to those observed in other studies [35] and resulted in improved performance under heat stress conditions. Further research is warranted to investigate these factors as well as to determine how the supplementation with *Ascophyllum nodosum* could affect gut health or other health factors in poultry, especially since *Ascophyllum nodosum* has been observed to have anti-viral, amplified phagocytic activity and anti-bacterial activity [14,15,16]. The mechanistic action of the reduced stress observed in this or other studies or the improved growth observed in other studies is not clear. This demands further investigation as well.

While the improvement or maintenance of growth and feed conversion is important to the poultry industry, it is not the only factor to consider. Heat stress detrimentally affects bird welfare, as maximizing animal welfare is becoming just as important a factor to consider in animal production, as society and agriculture focus more on it. The ability of *Ascophyllum nodosum* to reduce stress susceptibility, as observed in this current study, and others make it a viable alternative feed additive.

## 5. Conclusions

These results demonstrated that adding this seaweed extract to the feed of poultry can reduce their stress susceptibly even during a prolonged heat stress. Although it had no effect on the growth or feed conversion, it could still be used to improve the welfare of poultry during heat stress events.

## Figures and Tables

**Table 1:** Experimental diets and calculated nutrient content, by phase ^1^.

Ingredient, %	Starter (Day 1 to 14)	Grower (Day 15 to 28)	Finisher (Day 29 to 42)
Corn	58.4	63.1	68.95
Soybean meal (48% CP)	34.4	29.85	24.1
DL-Methionine	0.32	0.27	0.23
L-Lysine HCl	0.19	0.21	0.2
L-Threonine	0.06	0.07	0.07
Soy oil	2.85	2.85	3
Limestone	1.43	1.35	1.24
Monocalcium phosphate	1.56	1.44	1.29
Salt	0.43	0.37	0.23
Sodium bicarbonate	0	0.12	0.31
Vitamin and mineral premix	0.3	0.3	0.3
Calculated nutrients, %			
ME, kcal/kg	3057	3103	3169
Crude protein	22.07	20.26	17.94
Crude fat	5.4	5.53	5.84
Calcium	0.9	0.84	0.76
Total phosphorus	0.7	0.67	0.61
Av. phosphorus	0.45	0.42	0.38
Sodium	0.19	0.2	0.2

^1^ Vitamin premix contained 8818 IU vitamin A, 3086 IU vitamin D3, 37 IU vitamin E, 0.0132 mg B12, 4.676 mg riboflavin, 36.74 mg niacin, 16.17 mg d-pantothenic acid, 382.14 mg choline, 1.18 mg menadione, 1.4 mg folic acid, 5.74 mg pyridoxine, 2.35 mg thiamine and 0.44 mg biotin per kg diet and trace mineral premix contained 149.6 mg manganese, 125.1 mg zinc, 16.5 mg iron, 1.7 mg copper, 1.05 mg iodine, 0.25 mg selenium, a minimum of 6.27 mg calcium, and a maximum of 8.69 mg calcium per kg of diet. The carrier was calcium carbonate and the premix contained less than 1% mineral oil.

**Table 2:** Stress variables of broilers fed a control diet or one with a seaweed extract at 0.5 kg/metric ton either under normal temperatures or exposed to 14 days of heat stress.

Treatment	Corticosterone (pg/mL)	Heterophil/Lymphocyte Ratio	Asymmetry Score ^5^	HSP70 (pg/mL)
CNS ^1,3^	1080.5 ^b^	0.40 ^b^	1.52 ^b^	3.49 ^c^
SWNS ^2,3^	1085.2 ^b^	0.45 ^b^	1.49 ^b^	3.34 ^c^
CHS ^1,4^	2419.2 ^a^	1.05 ^a^	2.06 ^a^	4.33 ^a^
SWHS ^2,4^	1180.2 ^b^	0.61 ^b^	1.45 ^b^	3.60 ^b^
Diet effect				
C ^1^	1749.8	0.73	1.79	3.84
SW ^2^	1132.7	0.53	1.47	3.37
Heat stress effect				
NS ^3^	1082.9	0.63	1.50	3.46
HS ^4^	1799.7	0.83	1.75	3.75
SEM	152.0	0.06	0.06	0.04
*p* -value				
Main Effect diet	0.03	0.06	0.01	<0.001
Main Effect HS	0.01	<0.001	0.03	<0.001
Interaction	0.03	0.02	0.01	<0.001

^a,b,c^ Superscripts within column indicate significant differences (*p* < 0.05), ^1^ C = Control diet (*n* = 12), ^2^ SW = A diet supplemented with seaweed extract (0.5 kg/MT) (*n* = 12), ^3^ NS = Non-heat stressed (*n* = 6), ^4^ HS = Birds exposed to 35 °C for 16 h/day from 28 to 42 day of age (Heat Stressed) (*n* = 6), CNS ^1,3^, CHS ^1,4^, SWNS ^2,3^, SWHS ^2,4^, ^5^ (|L-R|Metatarsal length + |L-R|Metatarsal width + |L-R|Middle toe length)/3 = composite asymmetry score.

**Table 3:** Growth variables of broilers fed a control diet or one with a seaweed extract at 0.5 kg/metric ton, either under normal temperatures or exposed to 14 days of heat stress.

Treatment	D42 BW	d0–42 FCR	%CV	Uniformity Score
	(Kg)			
CNS ^1,3^	2.95 ^a^	1.602 ^c^	10.62	74.17
SWNS ^2,3^	2.91 ^a^	1.685 ^bc^	9.25	71.17
CHS ^1,4^	2.03 ^b^	1.89 ^a^	14.2	53.83
SWHS ^2,4^	2.16 ^c^	1.75 ^b^	15.3	53.83
Diet effect				
C ^1^	2.49	1.744	12.41	64
SW ^2^	2.53	1.719	12.28	62.5
Heat stress effect				
NS ^3^	2.93	1.643	14.2	58.83
HS ^4^	2.1	1.819	10.62	74.17
SEM	0.09	0.029	0.83	3.26
*p* -value				
Main	0.12	0.54	0.92	0.79
Effect diet				
Main	<0.001	<0.001	<0.001	0 < 0.001
Effect HS				
Interaction	0.04	0.02	0.38	0.79

^a,b,c^ Superscripts within column indicate significant differences (*p* < 0.05), ^1^ C = Control diet (*n* = 12), ^2^ SW = A diet supplemented with seaweed extract (0.5 kg/MT) (*n* = 12), ^3^ NS = Non-heat stressed (*n* = 6), ^4^ HS = Birds exposed to 35 °C for 16 h/day from 28 to 42 day of age (Heat Stressed) (*n* = 6), CNS ^1,3^, CHS ^1,4^, SWNS ^2,3^, SWHS ^2,4^.

## Data Availability

The data presented in this study are available on request from the corresponding author. The data are not publicly available due to privacy.
